# Enhancing Conductivity in 3D Organic Electrochemical Transistors with PEDOT–Tetramethacrylate Integration

**DOI:** 10.1021/acsmaterialslett.5c01170

**Published:** 2026-02-02

**Authors:** Viktorija Reinikovaite, İpek Sarıer, Martin Jönsson-Niedziółka, Nehar Celikkin, Marco Costantini, Marcin S. Filipiak

**Affiliations:** a State Research Institute Center for Physical Sciences and Technology, Vilnius 10257, Lithuania; b Institute of Physical Chemistry, Polish Academy of Sciences, Warsaw 01-224, Poland; c Centre for Advanced Materials and Technologies CEZAMAT, Warsaw University of Technology, Warsaw 02-822, Poland

## Abstract

Electroconductive hydrogels offer a unique combination of conductivity and biocompatibility, mimicking the extracellular matrix for bioelectronic applications. Herein, we present a homogeneous, conductive, and cytocompatible hydrogel-based organic electrochemical transistor (OECT) composite, combining methacrylated gelatin (GelMA), PEDOT:PSS, and tetramethacrylated PEDOT:TOS (PEDOT-TMA:TOS). This three-component hydrogel overcomes challenges in PEDOT aggregation, low conductivity, and cytotoxicity. The hydrogel exhibited remarkable electrochemical performance with a five-order impedance reduction, sheet resistance of 1.53 kΩ sq^–1^, and conductivity of 2.9 S m^–1^. OECTs fabricated with the hydrogel showed a threshold voltage of 0.216 V, transconductance of 2.1 mS, and an on/off ratio of 156.7. Live/dead assays confirmed excellent cytocompatibility due to efficient radical scavenging during cross-linking. This 3D conductive hydrogel network, compatible with cellular integration, establishes a foundation for next-generation bioelectronics, including sensors, neural interfaces, and tissue engineering.

## Graphical abstract

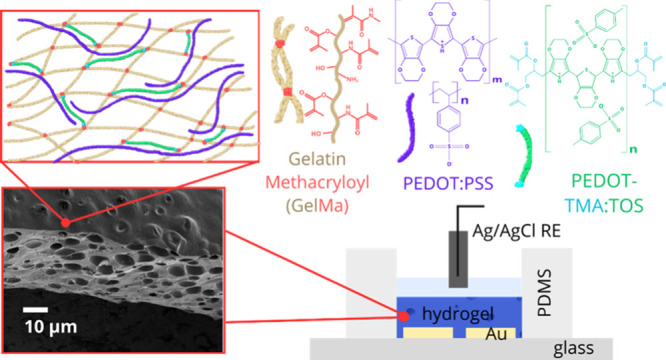

Electroconductive hydrogels represent an innovative class of biomaterials that integrate the hydrophilic and biocompatible nature of hydrogel matrices with the electrical conductivity of intrinsically (semi)­conducting polymers (CPs).^1^ This synergy allows these electroconducting hydrogels to mimic the properties of the extracellular matrix, making them flexible, highly hydrated, and capable of efficient electronic signal transduction.^2,3^ This unique combination renders them highly effective for a wide range of bioelectronics applications such as coatings on electrodes for monitoring^4^ and/or defibrillation,^5^ tactile sensors,^6,7^ soft actuators,^8^ and implantable neural electrodes.^9–11^ However, the diversity of available hydrogel matrices and CPs introduces both opportunities and challenges, requiring precise optimization of the material properties for specific applications.

The first synthesis of poly­(3,4-ethylenedioxythiophene) (PEDOT) doped with poly­(styrenesulfonate) (PSS) in 1991 marked a breakthrough in electronically conducting polymers.^12^ It was determined that hole-transporting (*p*-type) PEDOT:PSS dispersion in water possesses high conductivity, stability, transparency, and biocompatibility, thus, quickly gaining prominence in various industrial and research fields.^13^ The PSS-rich phase allows for ion and water uptake, while interconnected PEDOT-rich regions transport electronic charges.^14^ This unique microstructure that facilitates mixed conduction makes PEDOT:PSS an ideal material for usage in organic electrochemical transistor (OECT) technology, which requires efficient ion-to-electron transduction.^15^ However, incorporating PEDOT:PSS into hydrogels for bioelectronic applications does have certain limitations, i.e., achieving homogeneous dispersion while maintaining cytocompatibility. It has been reported that a 0.3% concentration of PEDOT:PSS in gelatin methacryloyl (GelMA) hydrogel can lead to aggregation and cytotoxicity, while only decreasing resistivity of the hydrogel by 1.7 times,^16^ highlighting the need for a careful balance in material composition.

Herein, we developed a highly homogeneous, conductive, and cytocompatible hydrogel-based OECT composite using GelMA derived from cold-water fish skin combined with commercially available PEDOT:PSS and a tetramethacrylated PEDOT derivative (PEDOT-TMA:TOS). The incorporation of PEDOT-TMA:TOS, which has not been previously used in cell or tissue culture applications, represents a novel enhancement to the material’s properties. This study provides proof-of-concept validation of the material’s electrochemical performance and cytocompatibility, enabling future flexible, biocompatible electronics.

In this study, PEDOT:PSS was selected as the CP due to its high stability, conductivity, and biocompatibility. Notably, PEDOT:PSS is the most extensively studied semiconductive polymer, particularly in the context of hydrogel preparation.^17^

Various modifications, including the use of different additives^18^ and dopants,^19^ and their influence on the performance of PEDOT-based-hydrogels have been thoroughly investigated.^17^ A commercially available source for the CP was chosen because of its high conductivity and purity compared to other available forms.^19^ The only necessary procedure prior to application is sonication at low temperatures (Figure 1a) to redisperse the CP-coiled chains. It was found that by simply mixing GelMA with PEDOT:PSS, hydrogels derived from cold-water fish skin gelatin uniquely minimize PEDOT:PSS aggregation. Notably, hydrogels made from cold-water fish skin gelatin possess several advantages over conventional gelatin A or B hydrogels, including lower gelation temperature^20^ and lower viscosity.^21^ Although the resulting fish GelMA lacks a certified Bloom strength and remains fluid even at 4 °C due to its very low proline/hydroxyproline content, we found that the same methacrylic-anhydride feed used for porcine or bovine GelMA (0.8 mL MA per 1 g of gelatin; target degree of substitution ≈75–85%) drives the reaction to an equally high degree of substitution. This is consistent with previous reports comparing fish and mammalian gelatins under identical conditions. Despite these benefits, the literature on fish GelMA hydrogels is limited. Currently, only a few papers explore such hydrogels, with just three papers incorporating PEDOT:PSS as CP.^16,22,23^ However, neither of the papers provides explicit reasoning for such a pairing. Moreover, the incorporation of PEDOT-TMA:TOS, which has not been previously used in such compositions, resulted in a homogeneous dispersion. A critical finding was that homogeneous hydrogel formation required adding both PEDOT solutions dropwise to the GelMA precursor solution under continuous vigorous vortexing. This level of agitation could only be achieved using a high-speed vortex mixer without silicone damping, where direct plastic-on-plastic contact generated intense agitation, while standard tabletop vortexers failed to provide sufficient mixing, leading to aggregation. The success of this method supports the hypothesis that PEDOT-TMA:TOS acts as a reactive oligomer,^24^ enabling instantaneous binding with PEDOT:PSS and GelMA. Notably, while the order of adding the two PEDOT solutions did not affect homogeneity, it was essential to add them to the vortexed GelMA solution rather than the reverse. When both PEDOTs were mixed, they precipitated out of their respective solvents and aggregated (data not shown). The short-term stability of the precursor solutions was assessed via centrifugation at 250 rpm for 1 min. This revealed sedimentation in both binary systems: GelMA/PEDOT-TMA:TOS (referred to as G/P-TMA) and GelMA/PEDOT:PSS (G/P). In contrast, the triple-component GelMA/PEDOT:PSS/PEDOT-TMA:TOS (G/P/P-TMA) mixture remained homogeneous (Supplementary Figure S2). Separately, visual inspection confirmed persistent aggregation during initial mixing of GelMA with PEDOT:PSS alone, corroborating literature reports that secondary sonication is required to achieve homogeneity.^16^ These complementary observations, specifically instability under centrifugation and aggregation during mixing, collectively point to PEDOT-TMA:TOS promoting synergistic interactions between GelMA and PEDOT:PSS (Figure 1b). We hypothesize that the methacrylation process may have altered the net charge of gelatin by reacting with amine side chain groups (histidine, arginine, glycine). This reaction would therefore reduce positive charges stemming from these side groups consequently lowering the isoelectric point.^25^ With a lower isoelectric point, we expect improved electrostatic repulsion among polymer chains and negatively charged components such as PEDOT:PSS, thereby reducing aggregation and enabling a more homogeneous and stable dispersion within the hydrogel network.

**Figure 1:**
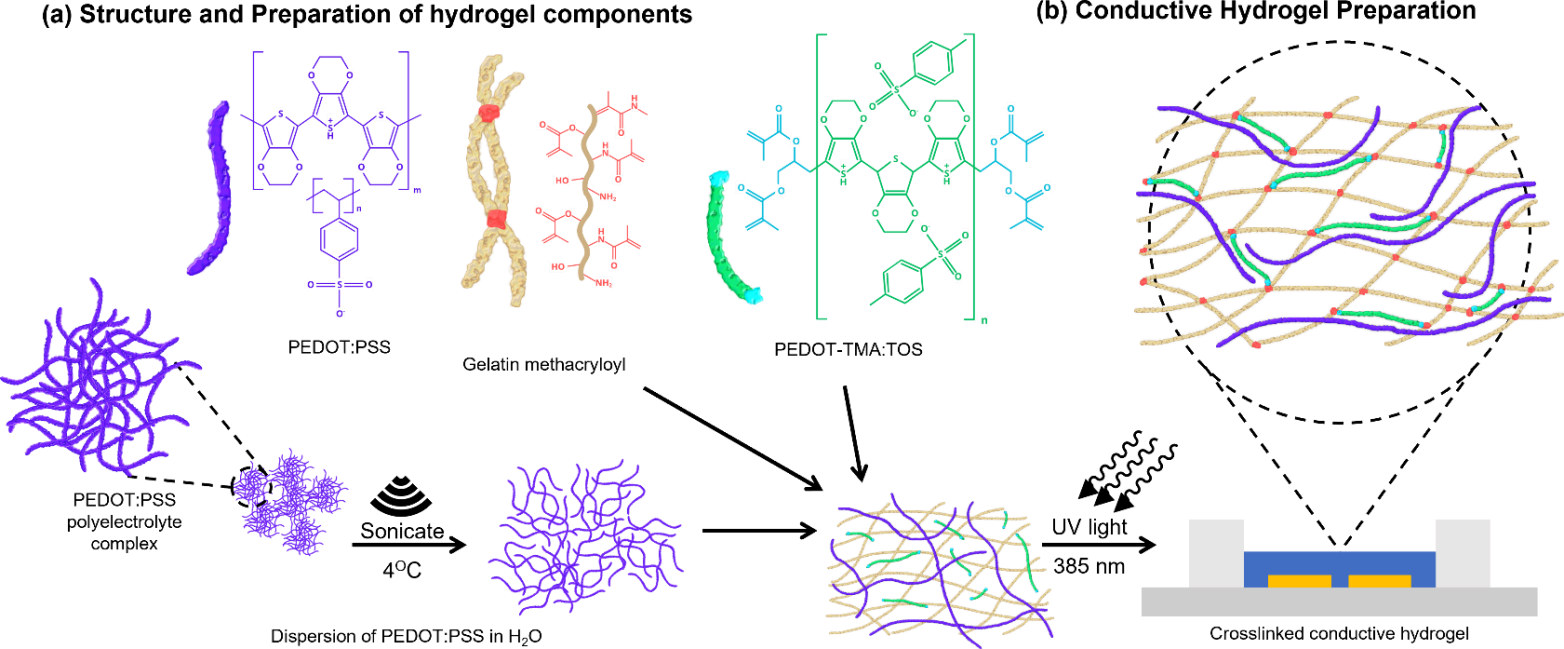
Preparation of the GelMA/PEDOT:PSS/PEDOT-TMA:TOS hydrogel. (a) Schematics of hydrogel component preparation and their molecular formulas. (b) Photopolymerization of the precursor solution to produce a cross-linked hydrogel network in a measuring chamber made of etched glass with two semicircle gold electrodes and PDMS sidewalls.

GelMA was photopolymerized with 0.4 wt % LAP under a 385 nm LED to yield self-supporting gels. LAP was chosen for its high molar absorptivity and aqueous solubility, enabling more reproducible gelation at lower light doses, consistent with prior GelMA reports.^26,27^ Steady-shear rheology of the uncross-linked GelMA/LAP precursor solutions (25 °C) showed clear non-Newtonian shear-thinning for all formulations, with viscosity decreasing monotonically as shear rate increased. Adding PEDOT:PSS substantially increased the low-shear viscosity and strengthened the shear-thinning response, whereas introducing PEDOT-TMA:TOS produced a more gradual viscosity decay and the highest viscosity at intermediate-to-high shear rates (Supplementary Figure S3). By testing different compositions, it was determined that 4% GelMA is the minimum concentration required to create a conducting hydrogel that is sufficiently gelated to be handled.^28^ The final composition of the conducting hydrogel that was developed in this paper is 4% GelMA mixed with 0.23% PEDOT:PSS and 0.11% PEDOT-TMA:TOS.

To assess chemical changes induced by photopolymerization and the incorporation of conductive components, ATR-FTIR spectra were collected for the hydrogels before and after UV curing (Figures S4–S5). To minimize effects of sample thickness and variable contact with the ATR crystal, spectra were normalized to the Amide I band (∼1645 cm^–1^), which arises from peptide backbone vibrations and is expected to remain stable during radical cross-linking of methacrylate groups. Quantitative comparison of normalized peak intensities (Supplementary Table S1) highlights clear differences between the conventional PEDOT:PSS formulation and the hybrid PEDOT-TMA:TOS system. In the three-component hydrogel (G/P/P-TMA), sulfonate-related bands at 1047, 1120, and 1178 cm^–1^ increased by 14–18% after curing, suggesting that methacrylate-functionalized PEDOT and the tosylate counterion promote exposure and stabilization of the conductive phase within the cross-linked network. By contrast, in the two-component hydrogel (G/P), the analogous sulfonate bands decreased by 10–20%, consistent with burial of unmodified PSS chains within the densifying mesh and/or phase reorganization that reduces their spectroscopic visibility. The hybrid system also exhibits a strong band at 1785 cm^–1^ (intensity ratio ∼ 1.0 vs Amide I) that remains essentially unchanged upon irradiation (+3.1%), which we attribute to residual propylene carbonate from the PEDOT-TMA formulation solvent. Finally, the 2981 cm^–1^ aliphatic C–H band decreased slightly (−7.2%), indicating that its prominence reflects overlapping tosylate/TMA contributions rather than the formation of new chemical species during curing.

Two-electrode electrochemical impedance spectroscopy (EIS) was used for initial screening, as it rapidly probes ionic and electronic transport and, thus, mixed conduction in the hydrogel prior to transistor fabrication. The device comprised two identical semicircular Au electrodes separated by a 300 μm gap, with the hydrogel cast in the mold (Figure 2a, inset). The Bode phase plot showed no significant difference between the three controls, where capacitive elements dominate at high frequencies and resistive elements dominate at low frequencies, which is highly consistent with the literature^16^ (Figure 2a). The G/P/P-TMA hydrogel exhibits a nearly constant phase angle close to 0° (−0.2 to −1.2°) over 10 mHz–500 kHz. Such a flat, near-zero phase response implies that the impedance is dominated by resistive rather than capacitive elements, consistent with a continuous percolation network of PEDOT domains bridging the gold electrodes and leaving little interfacial polarization to distort the signal. What is more, current and potential being almost in sync across the spectra is indicative of resistive elements being dominant with a minimal capacitive effect. The Bode total impedance spectrum shows slightly higher total impedance for the controls than is reported in the literature^16^ (Figure 2b). Importantly, all formulations were prepared and handled using the same sonication/mixing protocol described in the SI; therefore, comparisons between G/P and G/P/P-TMA reflect the effect of adding PEDOT-TMA:TOS under standardized preparation conditions. The three-component mixture had a decrease in total impedance by 5 orders of magnitude, and it was equally low throughout the entire frequency spectrum. Since the only difference between G/P and G/P/P-TMA samples was the addition of PEDOT-TMA:TOS, we attribute this significant decrease in resistivity to the increased cross-linked sites provided by the tetramethacrylate groups of PEDOT-TMA:TOS. The hydrogel sheet resistance was determined from EIS measurements. The sheet resistance (*R*_sheet_) was calculated using

1 $$R_{sheet}=R\left(\frac{W}{L}\right)$$

where *R* is the measured resistance, *W* is the electrode width, and *L* is the electrode length.

**Figure 2:**
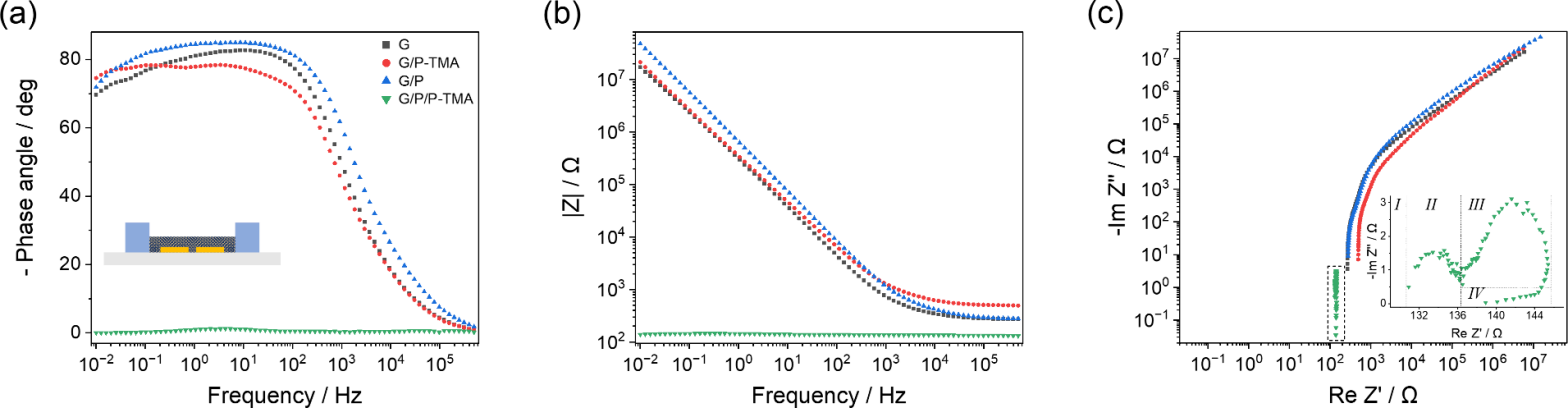
EIS studies of hydrogels composed of 4% GelMA (G), 4% GelMA with 0.23% PEDOT:PSS (G/P), and 4% GelMA with 0.23% PEDOT:PSS and 0.11% PEDOT-TMA:TOS (G/P/P-TMA). (a) Bode phase plot with an inset of the EIS cell schematic layout where two identical size gold electrodes serve as working and counter/reference electrodes and the hydrogel is cast inside the mold. (b) Bode total impedance plot. (c) Nyquist plot with a blowout of G/P/P-TMA hydrogel with highlighted effect zones: *I* – ionic resistivity of aqueous phase, *II* – ionic conductivity, *III* – electronic conductivity, and *IV* – unidentified function hook under the curve.

The volumetric conductivity (σ) was derived from

2 $$\sigma =\frac{1}{R_{sheet}\times t}$$

where *t* is the hydrogel thickness. With *t* = 225 μm, the sheet resistance was 1.53 kΩ sq^–1^ corresponding to a conductivity of 2.9 S m^–1^. Although higher conductivities have been reported for PEDOT:PSS-based hydrogels with values up to ∼40–50 S cm^–1^ (4000–5000 S m^–1^) for PEDOT:PSS-rich or processing-optimized hydrogels (e.g., using DMSO/ionic-liquid^29,30^ additives or templating/aggregation^31^ strategies), the electrical conductivity of the G/P/P-TMA hydrogel (2.9 S m^–1^) is sufficient for OECT operation. The comparatively modest conductivity observed here is consistent with the low PEDOT loading (0.23% + 0.11%) in the GelMA matrix and the absence of conductivity-enhancing post-treatments, and we therefore emphasize the performance achieved at low conductive-polymer content rather than absolute conductivity benchmarks (Supplementary Table S2).

The Nyquist plot revealed no significant difference among the three controls, whereas the triple mixture exhibited drastically lower real and imaginary impedance components, corresponding to reduced resistance and capacitance, respectively (Figure 2c). In the blowout, we see that the G/P/P-TMA has a higher real impedance value compared to the imaginary value, which correlates with phase angle findings. The complex Nyquist plot reveals four distinct regions: (*I*) A high-frequency intercept attributed to the ionic resistance of the aqueous phase within the cross-linked hydrogel network, consistent with its PBS-containing structure; (*II*) a semicircle at intermediate-higher frequencies, assigned to ionic charge transfer processes at the hydrogel/electrode interface; (*III*) a larger semicircle at midrange frequencies, dominated by electronic conduction through the PEDOTs network, as supported by the 5-order reduction in impedance compared to the PEDOT-free control; and (*IV*) a low-frequency hook (negative imaginary impedance) that does not cross the real axis. While similar features are observed in solar cell^32^ and corrosion systems^33^ (e.g., attributed to interfacial polarization or dynamic charge distribution), its origin here remains undetermined. The residual low-frequency curvature in the Nyquist trace can plausibly be ascribed to three well-documented phenomena: (i) finite-length ion diffusion within the PEDOT-rich domains, which adds a Warburg-type element and is frequently seen in OECTs when the ionic penetration depth approaches the channel thickness;^34^ (ii) slow viscoelastic relaxation of the hydrated polymer network at the PEDOT/hydrogel interface, giving rise to a constant-phase response;^35^ and (iii) pseudocapacitive volumetric charging of PEDOT:PSS, which typically produces a shallow semicircle with a 45° slope and a specific capacitance of 30–40 F cm^–3^.^36,37^ Reports of negative differential capacitance arising from mobile-ion reorganization exist, mainly in hybrid perovskites,^38^ but our spectra show neither an inductive loop nor a phase overshoot, making such contributions unlikely, albeit not yet conclusively excluded.

Figure 3 illustrates the structural analysis of the three-component G/P/P-TMA alongside its controls (G and G/P hydrogels) using scanning electron microscopy (SEM). The SEM micrographs demonstrate the two-component G/P hydrogel exhibited unevenly spread PEDOT:PSS aggregates, consistent with incomplete mixing as reported in prior studies^16^ (Figure 3b and Supplementary Figure S6a bright regions). The incorporation of additional PEDOT-TMA:TOS significantly enhanced homogeneity (Figure 3c), in contrast to other PEDOT:PSS/gelatin blends.^16^

**Figure 3:**
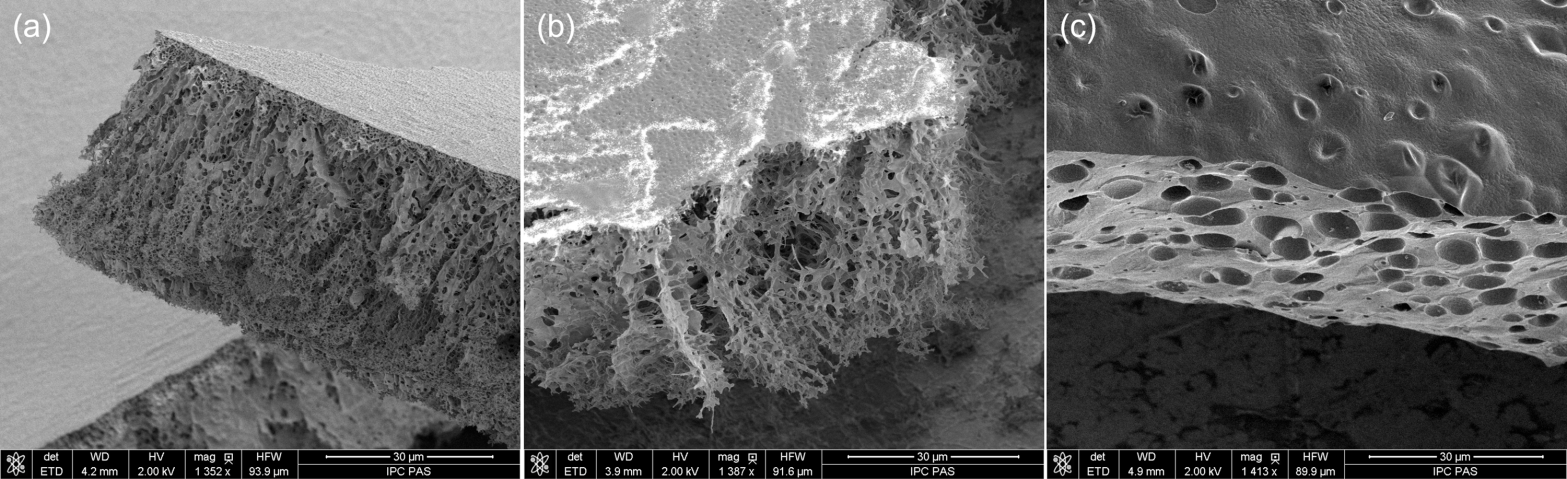
SEM images of lyophilized hydrogels: (a) 4% GelMA (G), (b) 4% GelMA with 0.23% PEDOT:PSS (G/P), and (c) 4% GelMA with 0.23% PEDOT:PSS and 0.11% PEDOT-TMA:TOS (G/P/P-TMA).

Notably, the three-component samples could be imaged without metal sputtering and remained stable even at an electron beam energy of 10 kV, with minimal artifacts or charging effects (Supplementary Figure S6b), which is consistent with increased intrinsic conductivity in the dried state. However, SEM was performed on lyophilized samples, and lyophilization/freezing can alter pore size, connectivity, and the apparent distribution of conductive domains; therefore, the observed morphology should be interpreted as a dried-state structure and may not quantitatively represent the hydrated network. Micrographs of G (Figure 3a) and G/P appear ragged and fibrous with no clearly defined cavities. In contrast, the G/P/P-TMA sample exhibited a bimodal porous architecture characterized by well-defined macropores (∼5–10 μm diameter) (Figure 3c) and a microstructured network of smaller pores (10–200 nm) (Supplementary Figure S6c,d). While such hierarchical porosity could support biological integration through macropore-enabled cell infiltration and transport via smaller pores, these functional implications should be considered qualitatively given the limitations of SEM on lyophilized hydrogels.

Cyclic voltammetry (CV) was used to assess the electrochemical properties of the G/P/P-TMA hydrogel. A custom cell with two identical semicircular Au electrodes at the bottom supported the cast hydrogel, with a Pt wire counter and Ag/AgCl reference electrode (Figure 4a). The CVs (Figure 4b) show capacitive behavior, with current scaling with the scan rate.

**Figure 4:**
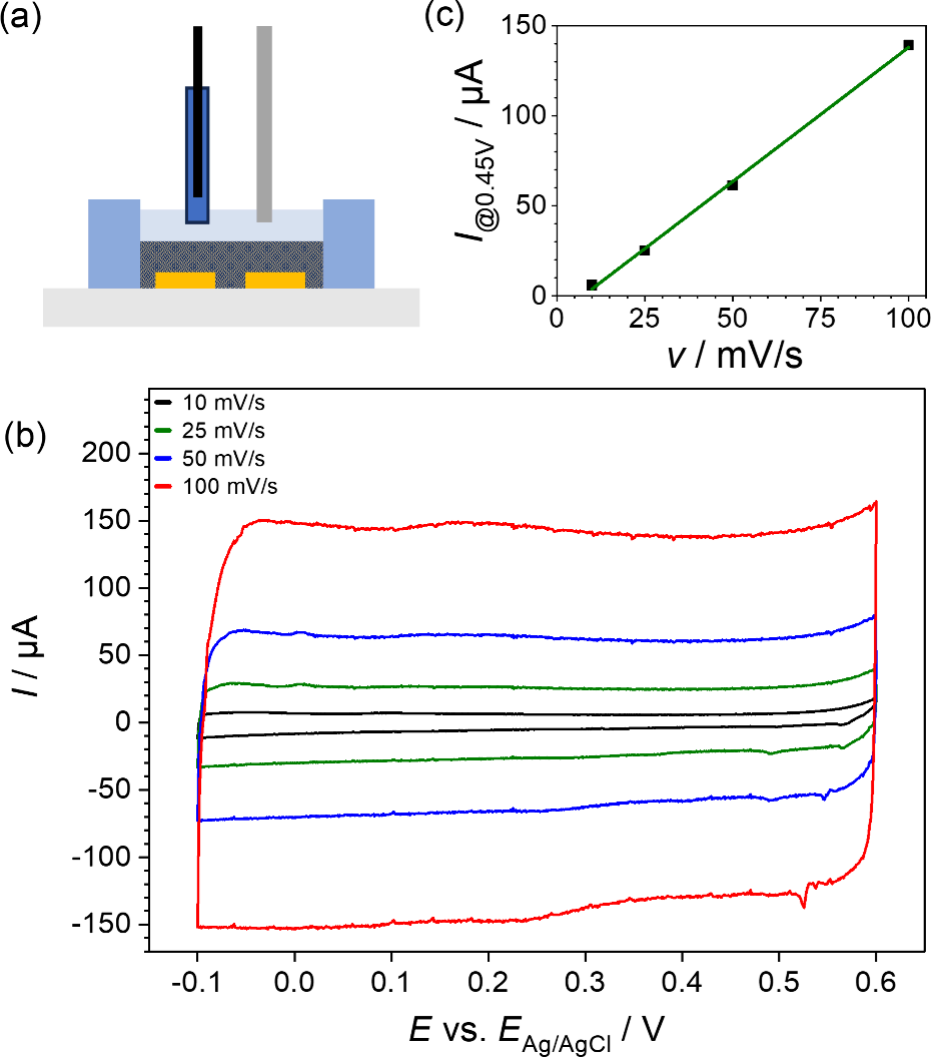
Testing of 4% GelMA with 0.23% PEDOT:PSS and 0.11% PEDOT-TMA:TOS (G/P/P-TMA) hydrogel was performed in (a) a cell containing two identical size semicircle gold electrodes, where the counter electrode was a Pt wire and the reference electrode was Ag/AgCl (KCl sat.). The cyclic voltammogram (b) shows current dependence of scan rate. The linear correlation (c) between current at 0.45 V and scan rate is indicative of capacitive behavior.

The linear relationship between the current (e.g., at 0.45 V) and the scan rate (Figure 4c) indicates that the hydrogel primarily charges its electrical double layer, reflecting efficient ionic interaction with the hydrogel surface. The total capacitance *C* of the hydrogel was determined from the CV data using the equation:

3 $$C=\frac{I}{\nu }$$

where *I* is the capacitive current (extracted at 0.45 V) and ν is the scan rate. While EIS confirms that the hydrogel is conductive throughout its volume, ionic charge injection during CV occurs only where the gel contacts a biased metal surface. In our setup, that active interface is the patterned bottom electrodes (∼9.6 mm^2^), not the entire gel/electrolyte boundary.

Therefore, we normalize by the electrode area *A* to report the true interfacial capacitance

4 $$C^{*}=\frac{C}{A}$$

The measured values were 1.49 mF for the total capacitance *C* and 15.5 mF cm^–2^ for the specific capacitance C*, reflecting the substantial electroactive surface area provided by the hydrogel’s structure.

OECTs utilizing the G/P/P-TMA hydrogel were fabricated and characterized to assess their performance as bioelectronic devices. The semiconducting hydrogel channel (*W* = 3.5 mm, *L* = 0.3 mm and *d* = 225 μm, giving *W*/*L* = 11.7) was drop-cast onto a planar electrode configuration consisting of source (S) and drain (D) semicircular electrodes (Figure 5 inset). Here, W denotes the effective channel width defined by the semicircular electrode overlap, and L corresponds to the interelectrode gap. Importantly, the relatively thick, 3D channel (*d* = 225 μm) and semicircular electrode geometry differ from the thin-film OECT architectures most reported; consequently, ionic penetration dynamics and characteristic time constants may differ, and the discussion below focuses on quasi-steady-state transfer characteristics. This configuration enabled robust modulation of the charge transport within the 3D conductive network of the hydrogel. Transfer curves confirmed transistor operation in the three-component hydrogel (channel opening/closing), whereas two-component controls showed only capacitive electrical-double-layer charging. The key performance metrics of the OECTs based on the G/P/P-TMA hydrogel included a threshold voltage *V*_th_ = 0.216 ± 0.031 V, a maximum transconductance *g*_m,MAX_ = 2.13 ± 0.86 mS, and an on/off current ratio *I*_ON_/*I*_OFF_ = 156.69 ± 61.62. To enable a geometry-normalized comparison with literature reports, we additionally report the geometry-normalized transconductance *g*_m,norm,MAX_ = *g*_m,MAX_ × *L*/(*W* × *d*), yielding *g*_m,norm,MAX_ 8.1 ± 3.3 mS cm^–1^. These values are consistent with or exceed those reported for PEDOT:PSS-based OECTs in the literature^39^ noting that direct comparison of absolute *g*_m,MAX_ values without geometry normalization should be treated with caution given the markedly different channel thickness and device geometry (Supplementary Table S2). The hydrophilic and 3D nature of the hydrogel also supports seamless integration with biological systems as its interconnected pore structure accommodates biological cells while maintaining excellent electrochemical properties.

**Figure 5:**
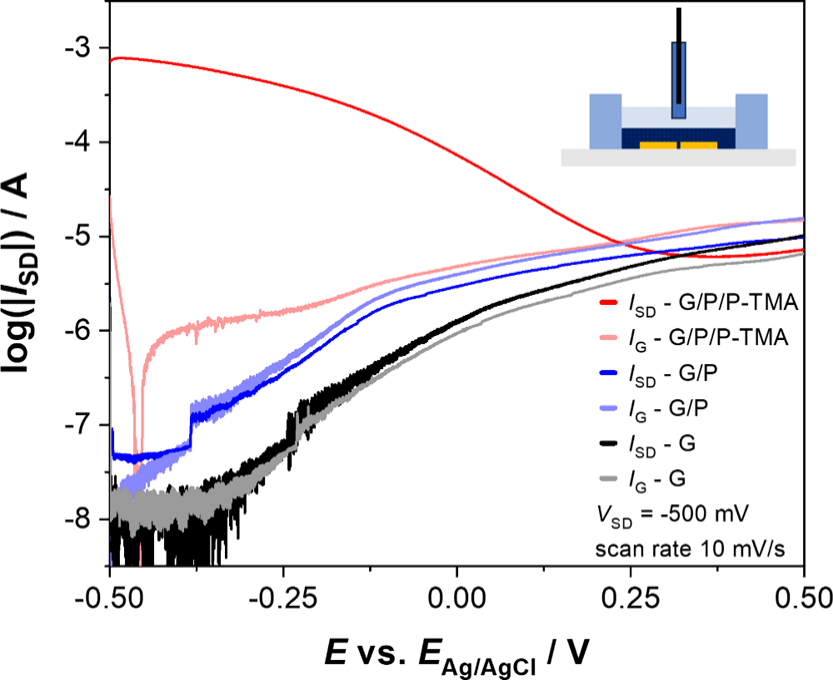
Organic electrochemical transistor (OECT) prepared with different compositions of hydrogel: transfer curves for the 3-component mixture (G/P/P-TMA) vs. the controls (2 components—G/P and only G).

Finally, the cytocompatibility of G/P/P-TMA was assessed by a live/dead assay (Supplementary Figure S7). Viability differed significantly across formulations (*p* < 0.001): G/P/P-TMA showed >98% viable cells, higher than G/P (∼68%) and GelMA (∼25%) (*p* < 0.001). The improvement is attributed to more complete methacrylate cross-linking and radical scavenging during photopolymerization, whereas incomplete GelMA cross-linking likely left cytotoxic unreacted methacrylated groups, consistent with reports that LAP itself is noncytotoxic.^26^ The results confirm that the three-component G/P/P-TMA hydrogel mitigates cytotoxic effects associated with methacrylated groups and provides a biocompatible environment at all time points (>98% cell viability). Together with its improved electrochemical performance, this supports its use in cytocompatibility-critical bioelectronic interfaces, including biosensors, neural interfaces, and tissue-engineering platforms.

Here, we developed a homogeneous, conductive, and cytocompatible hydrogel-based OECT composite by combining methacrylated fish gelatin (GelMA), PEDOT:PSS, and tetramethacrylated PEDOT:TOS (PEDOT-TMA:TOS). This formulation addresses key limitations of PEDOT-based hydrogels, including PEDOT aggregation, limited conductivity, and cytotoxicity. Incorporation of PEDOT-TMA:TOS improved conductivity (sheet resistance 1.53 kΩ sq^–1^) and reduced impedance by 5 orders of magnitude (EIS). SEM analysis confirmed a uniform dispersion of the conductive components within the GelMA matrix without phase separation. In 3D OECTs, the hydrogel enabled active channel modulation with *V*_th_ = 0.216 ± 0.031 V, *g*_m,MAX_ = 2.13 ± 0.86 mS (*g*_m,norm,MAX_ = 8.1 ± 3.3 mS cm^–1^), and an on/off ratio of 156.69 ± 61.62, indicating its potential for flexible sensors, soft actuators, and implantable devices. Future work would focus on stability and expanded *in vitro*/*in vivo* validation as well as broader device functionality.

## Supplementary Material



## Notes

Materials and methods, hydrogels preparation and characterization, and supplementary figures and comparison tables (Figures S1–S7 and Tables S1 and S2) (PDF)

## ABBREVIATIONS

PEDOT: poly­(3,4-ethylenedioxythiophene)
PSS: polystyrenesulfonate
PEDOT-TMA: poly­(3,4-ethylenedioxythiophene)-tetramethacrylate
GelMA: gelatin methacryloyl
SEM: scanning electron microscopy
CV: cyclic voltammetry
EIS: electrochemical impedance spectroscopy
OECT: organic electrochemical transistor
LAP: lithium phenyl-2,4,6-trimethylbenzoylphosphinate
MA: methacrylate
