# Supplementary material for: Enhancing Conductivity in 3D Organic Electrochemical Transistors with PEDOT–Tetramethacrylate Integration
Source: ACS Mater Lett. 2026 Feb 2;8(3):750–6. doi: 10.1021/acsmaterialslett.5c01170 (PMC13308696; doi:10.1021/acsmaterialslett.5c01170)
Supplement: Supplementary file 1 [file tz5c01170_si_001.pdf]

Supplementary Information

Enhancing Conductivity in 3D Organic Electrochemical Transistors with PEDOT-tetramethacrylate Integration

Viktorija Reinikovaite[1,2], İpek Sarıer[3], Martin Jönsson-Niedziółka[2], Nehar Celikkin[2], Marco Costantini[2], and Marcin S. Filipiak[2,3,*]

[1] State Research Institute Center for Physical Sciences and Technology, Vilnius, 10257, Lithuania

[2] Institute of Physical Chemistry, Polish Academy of Sciences, Warsaw, 01-224, Poland

[3] Centre for Advanced Materials and Technologies CEZAMAT, Warsaw University of Technology, Warsaw, 02-822, Poland

[*] Corresponding author, to whom correspondence should be addressed E-mail: Marcin S. Filipiak marcin.filipiak@pw.edu.pl

Table of Contents

1. Methods and Materials 3
 1.1 Chemicals 3
 1.2 Synthesis of GelMA and its hydrogels 3
 1.3 Gold electrode preparation 4
 1.4 Electrochemical measurements 4
 1.5 Scanning Electron Microscopy 6
 1.6 Cell cytotoxicity test 6
 1.7 Gel fraction/swelling 7
 1.8 Rheology 8
 1.9 FTIR 8
 1.10 Statistical analysis 9
2. Supplementary Figure S2. Short-term stability of precursor solutions 10
3. Supplementary Figure S3. Rheology 11
4. Supplementary Figure S4 and S5 + Table S1 . FTIR of the hydrogels before and after UV crosslinking 12
5. Supplementary Figure S6. Hydrogel surface morphology 15
6. Supplementary Figure S7. C2C12 Live/Dead assay 16
7. Supplementary Table S2. Different approaches to GelMA + CP composition and resulting properties 18
References 19

1. Methods and Materials

1.1 Chemicals

Gelatin from cold water fish skin, methacrylic anhydride, 2-hydroxy-4′-(2-hydroxyethoxy)-2-methylpropiophenone (Irgacure 2959), lithium phenyl-2,4,6-trimethylbenzoylphosphinate (LAP), hexamethyldisilazane (HMDS), xylene, potassium hexacyanoferrate(II/III), and poly(3,4-ethylenedioxythiophene) tetramethacrylate end-capped solution, 0.5 wt. % (dispersion in propylene carbonate), containing p-toluenesulfonate as dopant (PEDOT-TMA:TOS) were purchased from MERCK (St Louis, MO, USA). Poly(3,4-ethylenedioxythiophene):polystyrene sulfonate (PEDOT:PSS) was in the form of an aqueous dispersion called Clevios PH 1000 and was purchased from Heraeus (Hanau, Germany). The ma-N415 photoresist and ma-D532/s developer purchased from MicroChemicals (Ulm, Germany). Calcein AM and ethidium homodimer-1 for LIVE/DEAD Cell Imaging Kit were purchased from ThermoFisher (Waltham, MA). Dulbecco's modified phosphate buffer saline (DPBS) with no calcium or magnesium, phosphate buffer saline (PBS, pH 7.4), Dulbecco's modified eagle medium (DMEM), fetal bovine serum (FBS), trypsin, penicillin and streptomycin purchased from GE Healthcare Life Sciences (Logan, Utah). Pt wire (5 N, *Ø* 0.5 mm) and Au disc (5N) were from the Mint of Poland. Water was filtered and deionized with a Sartorius Arium Comfort I system.

1.2 Synthesis of GelMA and its hydrogels

Photo-crosslinkable GelMA pre-polymer with ~ 80 % methacryloyl functionalization degree, was first synthesized by adding 10 g of gelatin from cold-water fish skin to 100 mL of Dulbecco's phosphate-buffered saline (DPBS) at 60°C. Once fully dissolved, 8 mL of methacrylic anhydride were added dropwise to the solution and allowed to homogenize. After 3 h of reaction, 300 mL of pre-heated DPBS at 60°C were added to the solution to stop the reaction. The mixture was dialyzed in a 12 – 14 kDa cutoff dialysis tubing against the deionized (DI) water for one week, replacing the DI water twice daily. Following dialysis, the solution was centrifuged at 4,000 rpm for 10 min to remove precipitates. The supernatant was then lyophilized for 7 days in a Benchtop Freeze Dryer (Labconco, MO, USA) with a condenser temperature of -80°C. The resulting fish GelMA prepolymer was stored at -20°C until use.

To form the hydrogels, 4% (w/v) GelMA pre-polymer was dissolved in PBS containing 0.4% (w/v) photo-initiator (note that the photoinitiator 4% stock solution was prepared in ethanol

and further diluted 10 times), a concentration known to be nontoxic to cells encapsulated within GelMA hydrogels[1]. Commercially available PEDOT:PSS (Clevios PH 1000) solution due to its tendency to aggregate was sonicated for 30 min at 4°C with SONOPULS ultrasonic homogenizer (Bandelin electronic GmbH & Co. KG, Berlin, Germany) set to 70W power, 10 sec on/off pulses. Then Clevios PH1000 and PEDOT-TMA:TOS (Oligotron) solutions were sterile filtered (0.22 μm) to remove insoluble aggregates. PEDOTs were added to solutions of GelMA to make 0.23% of final volume. Control GelMA sample with 0.23 % PEDOT:PSS and GelMa with no PEDOTs were also prepared. Finally, the prepared hydrogels crosslinking was initiated with UV irradiation (385 nm at 1.69 mW cm^{-2}) for 3.5 min.

1.3 Gold electrode preparation

Clean 25 mm × 50 mm × 1.1 mm glass plates (Delta Technologies, Loveland, CO, USA) were first treated with Zepto air plasma (Diener Electronics GmbH & Co. KG, Ebhausen, Germany) for 10 min and dehydrated at 200°C for 20 min. A 10% (v/v) solution of hexamethyldisilazane (HMDS) in xylene was then spin-coated onto the activated glass surfaces at 3000 rpm for 30 sec using a WS-650MZ-23NPPB spin coater (Laurell Technologies Corporation, Lansdale, PA, USA), followed by solvent evaporation at 95°C for 1.5 min. Subsequently, ma-N415 photoresist was applied via spin-coating (3000 rpm, 30 sec) and soft-baked at 95°C for 1.5 min. The photoresist was UV-patterned through a mask for 100 sec (λ = 365 nm, 831 mW/cm^2 intensity) using MJB4 mask aligner (SÜSS MicroTec, Germany). Developed under constant vortexing in ma-D532/s for 8.5 min, the slides were rinsed thoroughly with DI water trice and dried under nitrogen gas stream. Finally, the substrates were sputter-coated with a 10-nm titanium adhesion layer and a 100-nm gold layer (deposition rate: Ti 0.06 nm s^{-1}, Au 0.1 nm s^{-1}, base pressure: 1.1 x 10^{-3} mbar) with Leica EM MED020 (Leica, Copenhagen, Denmark) modular high vacuum coating system.

1.4 Electrochemical measurements

All the electrochemical measurements unless stated otherwise were performed with a PalmSens4 potentiostat/galvanostat and controlled via PSTrace 5.8.1704 software, while OECT measurements were done with EmStat Pico bipotentiostat (PalmSens BV, Houten, the Netherlands). The three-electrode electrochemical cell consisted of a platinum wire counter electrode, DRI-REF2 Ag/AgCl (3 M KCl) (World Precision Instruments GmbH, Germany)

reference electrode, and two identical size semicircle Au working electrodes (preparation described in 1.3 section, Figure S1).

Electrochemical impedance spectroscopy (EIS) measurements were conducted at the open-circuit potential at ambient temperature (23 ± 1°C). To ensure consistent hydrogel geometry during measurements, a custom-fabricated polydimethylsiloxane (PDMS) mold was positioned atop the gold working electrode, maintaining a fixed hydrogel thickness of 225 μm. The frequency sweep spanned 0.01 Hz to 500 kHz (logarithmically distributed, 10 points per decade) with an applied AC perturbation of 10 mV, achieving steady-state conditions at each frequency (validated by less than 2% deviation in consecutive scans). Prior to measurements, the system stability was verified through open-circuit potential monitoring until potential drift fell below 1 mV/min.

The performance of the fabricated hydrogels was studied using the cyclic voltammetry (CV) technique with 5 mM equimolar potassium hexacyanoferrate(II/III) ($K_4[Fe(CN)_6]/K_3[Fe(CN)_6]$) in 1× PBS (pH 7.4), prepared fresh each time. Parameters for CV were as follows: scan window set to -0.1V to 0.6V, varying scan rate from 0.01 to 0.1 V s^{-1}, step potential 0.005 V. Gold electrodes were sequentially cleaned with ethanol and deionized water, then dried under nitrogen, enabling reuse across multiple measurement cycles.

For the OECT measurements, the two Au electrodes were connected separately as source and drain contacts to the PalmSens' EmStat Pico bipotentiostat (for details of connection, see reference[2]) and an additional Ag/AgCl reference electrode (same as previously) worked as an electrochemical gate. The gate voltage V_g was swept from -0.5 to 0.5 V, whereas the source-drain voltage V_{SD} was kept constant at -0.5 V.

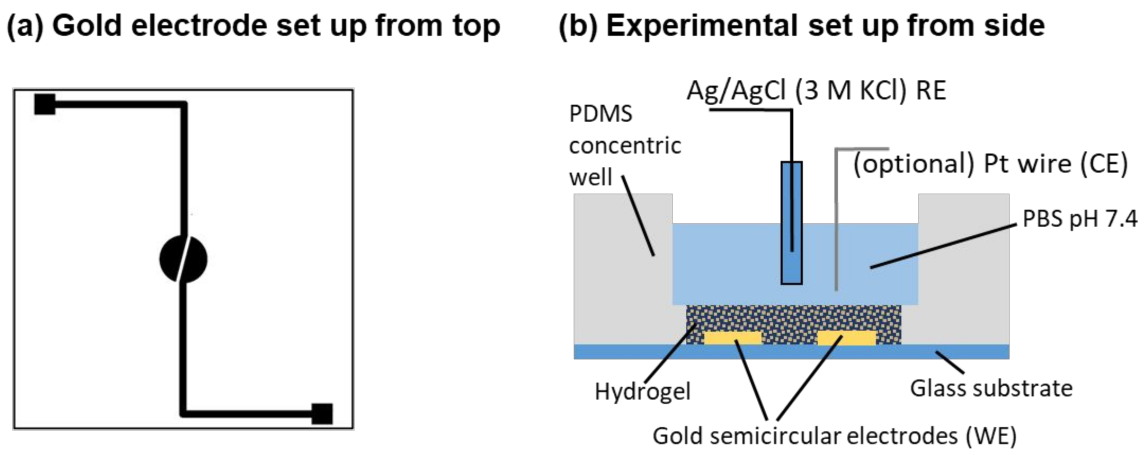

Figure S1. *Prepared gold electrode layout. (a) View from the top showing two identical semicircles that depending on the experiment can be used in 2-electrode system (WE and CE/RE, EIS case), in 3-electrode set up (combined as WE for electrochemical mode), or in FET set-up (source and drain for Organic Electrochemical Transistor mode). (b) Exemplary 3-electrode set up where two gold semicircles are combined into one WE electrode.*

1.5 Scanning Electron Microscopy

The internal microstructures of photo-cured hydrogels were analyzed by scanning electron microscopy (SEM). After crosslinking, the hydrogels were snap frozen in liquid nitrogen. Then the samples were placed in the K775X turbo freeze dryer (Quorum Technologies, Madrid, Spain) and lyophilized for 48 h. Due to anticipated low electrical conductivity the GelMa and GelMa with PEDOT:PSS samples were sputter coated with 10 nm of gold with Leica EM MED020 (Leica, Copenhaven, Denmark) modular high vacuum coating system. SEM micrographs of the hydrogels were captured using an FEI Nova Nano – SEM 450 (Regen Microscopy, France) at 2.0kV and 5-7 nm working distance.

1.6 Cell cytotoxicity test

C2C12 cell lines came from the American Type Culture Collection (ATCC, Manassas, USA) and were cultured under standard conditions (37°C; 5% CO_2) with Dulbecco's modified eagle medium (DMEM) supplemented with 10% fetal bovine serum and 1% penicillin/streptomycin. To evaluate hydrogel cytotoxicity, the 4 % hydrogel precursors with or without PEDOTs were prepared as previously described. Prior to mixing all components were sterile filtered with 22 µm filters to ensure sterility. Cells were detached using trypsin-EDTA (0.25% v/v), centrifuged and resuspended in the precursor solution at 1×10^5 cells mL^{-1} before precursor crosslinking. To avoid hydrogel drying during crosslinking, a humidifying chamber was used. Samples were cultured in 96 well plates with 400 µL of media. Medium was changed twice in the first 30 min after crosslinking to remove unreacted photoinitiator and subsequently changed daily. Cell viability was assessed on days 1, 3, and 7. For LIVE/DEAD assay (L3224, ThermoFisher, Waltham, MA), cells were stained with calcein AM (live) and ethidium homodimer-1 (dead) and incubated for 30 min at room temperature. Fluorescent imaging was performed on a Nikon A1 HD25 confocal and multiphoton microscope (Nikon Instruments

Inc., NY, USA). Cell viability was quantified using ImageJ software (NIH) from LIVE/DEAD images.

1.7 Gel fraction/swelling

Swelling behavior of 4% GelMA, 0.4% LAP, 0.23% PEDOT:PSS, and 0.11% PEDOT:TMA hydrogel was evaluated to determine water uptake and crosslinking efficiency. Immediately after UV crosslinking, each hydrogel sample was gently blotted to remove surface water and weighed to obtain the initial wet weight (W_0). Samples were then immersed in PBS and incubated overnight to reach equilibrium swelling. The swollen hydrogels were removed from PBS, briefly dried with tissue to remove excess surface liquid, and weighed to obtain the swollen weight (W□). To determine the polymer content that remained after swelling/extraction, the gels were subsequently freeze-dried for 24 h and weighed again to obtain the dry weight (Wd). This post-swelling dry weight represents the mass of the crosslinked polymer network after removal of the soluble fraction.

The gel fraction averaged 0.0486 ± 0.0093, indicating that approximately 5 % of the initial polymer mass remained as an insoluble, crosslinked network after extraction. The swelling ratio, calculated as $(W_s - W_d)/W_d$, was 25.97 ± 3.72, consistent with a highly hydrated hydrogel structure.

These results demonstrate that PEDOT-TMA incorporation leads to a stable crosslinked network capable of substantial water uptake, which supports our interpretation of covalent integration within the GelMA matrix.

**Sample**	W_0 **[mg]**	W_d **[mg]**	W_s **[mg]**	**Gel Fraction (W_d/W_0)**	**Swelling Ratio ($(W_s - W_d)/W_d$)**
1	70.6	3.0	78.0	0.0425	26.0
2	71.6	2.7	81.8	0.0377	30.30
3	73.8	3.9	90.0	0.0528	23.08
4	75.0	3.7	90.3	0.0493	24.41
5	65.4	4.2	90.6	0.0642	21.57
6	66.3	3.0	91.4	0.0452	30.47
**Mean ± SD**	—	—	—	**0.0486 ± 0.0093**	**25.97 ± 3.72**

Swelling Ratio (SR)

$$SR = \frac{W_s - W_d}{W_d}$$ where

W_s= swollen weight (after equilibrium immersion in water),

W_d= dry weight (after drying).

The SR expresses how many times the dry material's mass increases due to water uptake, quantifying hydration capacity.

Gel Fraction (GF)

$$GF = \frac{W_d}{W_0}$$ where

W_0= initial wet weight before extraction,

W_d= final dry weight after extraction/drying.

The GF represents the insoluble, crosslinked portion of the hydrogel network remaining after solubilization and extraction.

1.8 Rheology

Dynamic and steady-shear rheological measurements were performed using a rotational rheometer (Kinexus Pro+, Malvern Instruments, UK). A stainless-steel cone–plate geometry (CP1/50, 1° cone angle, 50 mm diameter; geometry ID: SR0491 SS) was used for all experiments. Approximately 1 mL of each hydrogel formulation was loaded onto the lower plate, and the cone was lowered to a working gap of 0.050 mm. Excess material was trimmed prior to testing. All measurements were conducted at 25 °C.

1.9 FTIR

FTIR measurements were performed using a Vertex 80v spectrophotometer (Bruker Inc., USA) equipped with a Platinum ATR accessory (diamond crystal). Spectra were recorded in the range of 4000–400 cm^{-1} (4 cm^{-1} resolution, 64 scans) and normalized to the Amide I band (~1645 cm^{-1}).

1.10 Statistical analysis

All experiments included at least three independent biological replicates. Data are presented as mean ± standard deviation (SD). Statistical analysis was performed using OriginPro 2021 (Version 9.8.0.200). For live/dead assay data, a two-way ANOVA with Tukey's HSD post-hoc test assessed effects of hydrogel composition and time, with biological replicates as experimental units. Significance was defined as $p < 0.05$.

2. Supplementary Figure S2. Short-term stability of precursor solutions

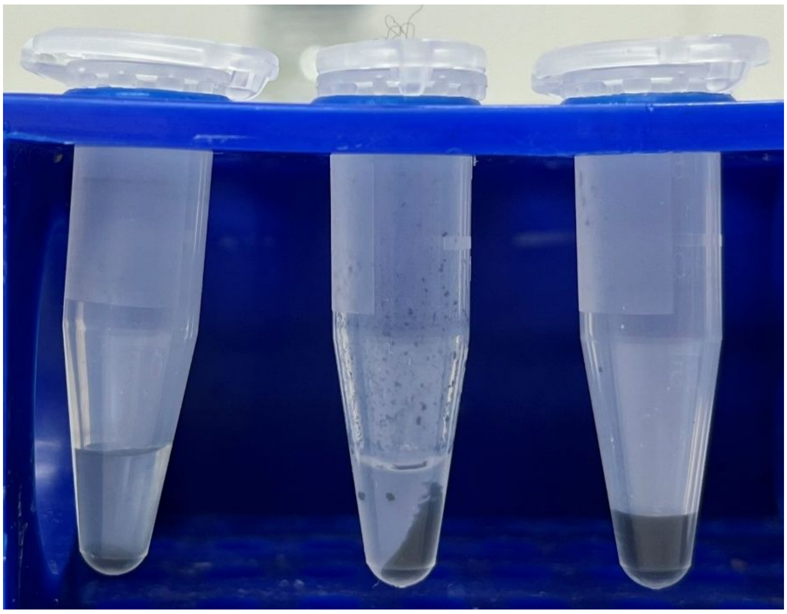

Figure S2. *Short-term stability assessment of pre-crosslinking precursor solutions following centrifugation at 250 RPM for 1 minute. Left to right: GelMA/PEDOT-TMA:TOS (G/P-TOS) showing sedimentation, GelMA/PEDOT:PSS (G/P) with visible phase separation, and homogeneous GelMA/PEDOT:PSS/PEDOT-TMA:TOS (G/P/P-TOS) demonstrating superior dispersion stability.*

3. Supplementary Figure S3. Rheology

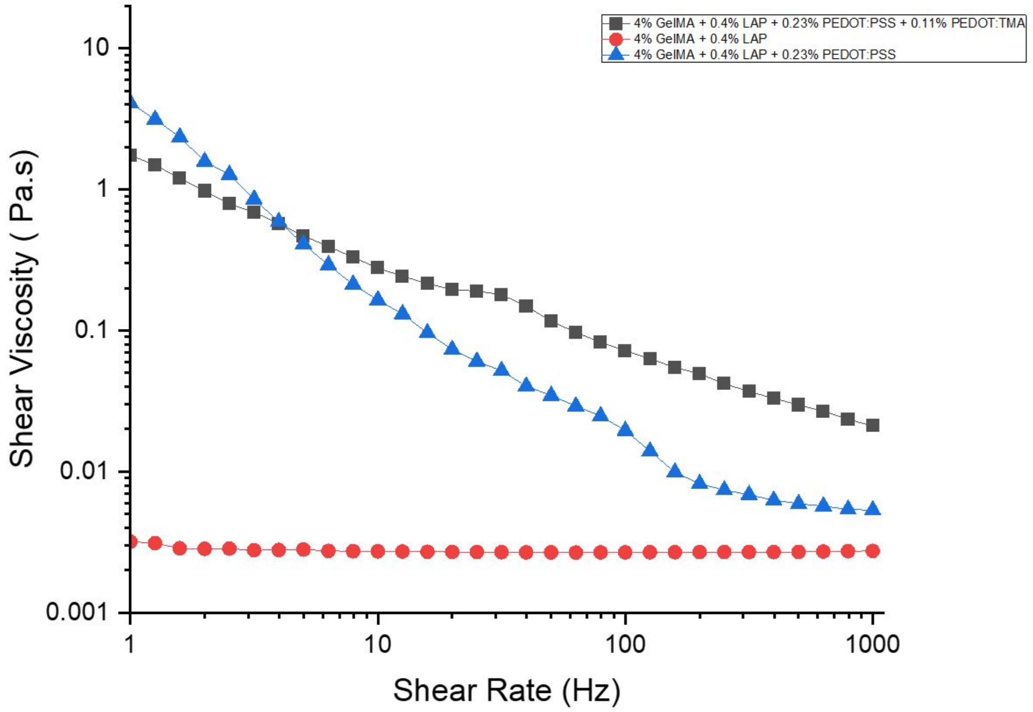

Figure S3. *Rheology results showing the shear viscosity profiles of 4% GelMA + 0.4% LAP, 4% GelMA + 0.4% LAP + 0.23% PEDOT:PSS, and 4% GelMA + 0.4% LAP + 0.23% PEDOT:PSS + 0.11% PEDOT:TMA across increasing shear rates.*

4. Supplementary Figure S4 and S5 + Table S1 . FTIR of the hydrogels before and after UV crosslinking

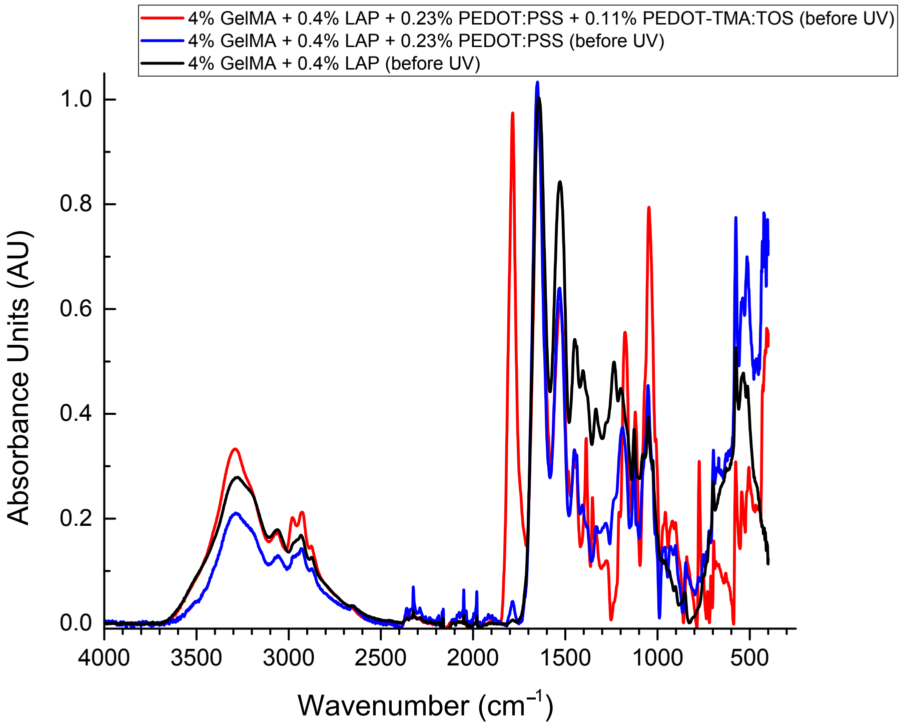

Figure S4. *FTIR spectra of the hydrogel precursor solutions prior to photopolymerization (Before UV). Spectra were acquired for the hybrid GelMA/PEDOT:PSS/PEDOT-TMA:TOS system (3-part), the standard GelMA/PEDOT:PSS system (2-part), and the GelMA Control.*

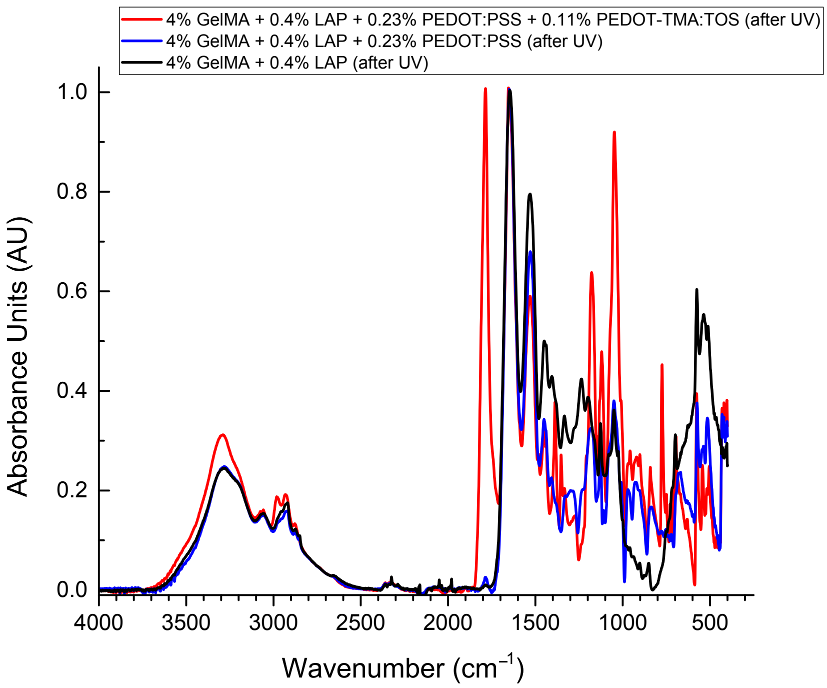

***Figure S5**. FTIR spectra of the crosslinked hydrogel networks (After UV).*

Table S1. *Quantitative analysis of FTIR peak intensity ratios normalized to the Amide I internal standard (1645 cm⁻¹) for the hybrid (3-part) and standard (2-part) hydrogel systems before and after UV photopolymerization. The data highlights the relative increase in sulfonate dopant exposure in the PEDOT-TMA:TOS-containing system, contrasted with a decrease in the standard PSS-only system.*

**Sample**	**Wavenumber (cm^{-1})**	**Assignment**	**Ratio (Before UV)**	**Ratio (After UV)**	**Change (%)**
**GelMA + PEDOT:PSS + PEDOT-TMA:TOS**	1047	Sulfonate sym. stretching (νs. SO_3^-)	0.790	0.912	**+15.5**
	1120	Sulfonate / Matrix overlap	0.401	0.475	**+18.3**
	1178	Sulfonate asym. stretching (νas SO_3^-)	0.552	0.632	**+14.5**
	1785	C=O stretching (cyclic carbonate)*	0.969	0.999	**+3.1**
	2981	Aliphatic C–H stretching	0.200	0.186	-7.2
**GelMA + PEDOT:PSS**	1051	Sulfonate sym. stretching (νs SO_3^-)	0.440	0.378	**-14.0**
	1128	Sulfonate / Ether overlap	0.347	0.280	**-19.5**
	1188	Sulfonate asym. stretching (νas SO_3^-)	0.362	0.322	**-10.9**

* The peak at 1785 cm^{-1} is attributed to residual propylene carbonate solvent in the PEDOT-TMA:TOS formulation.

5. Supplementary Figure S6. Hydrogel surface morphology

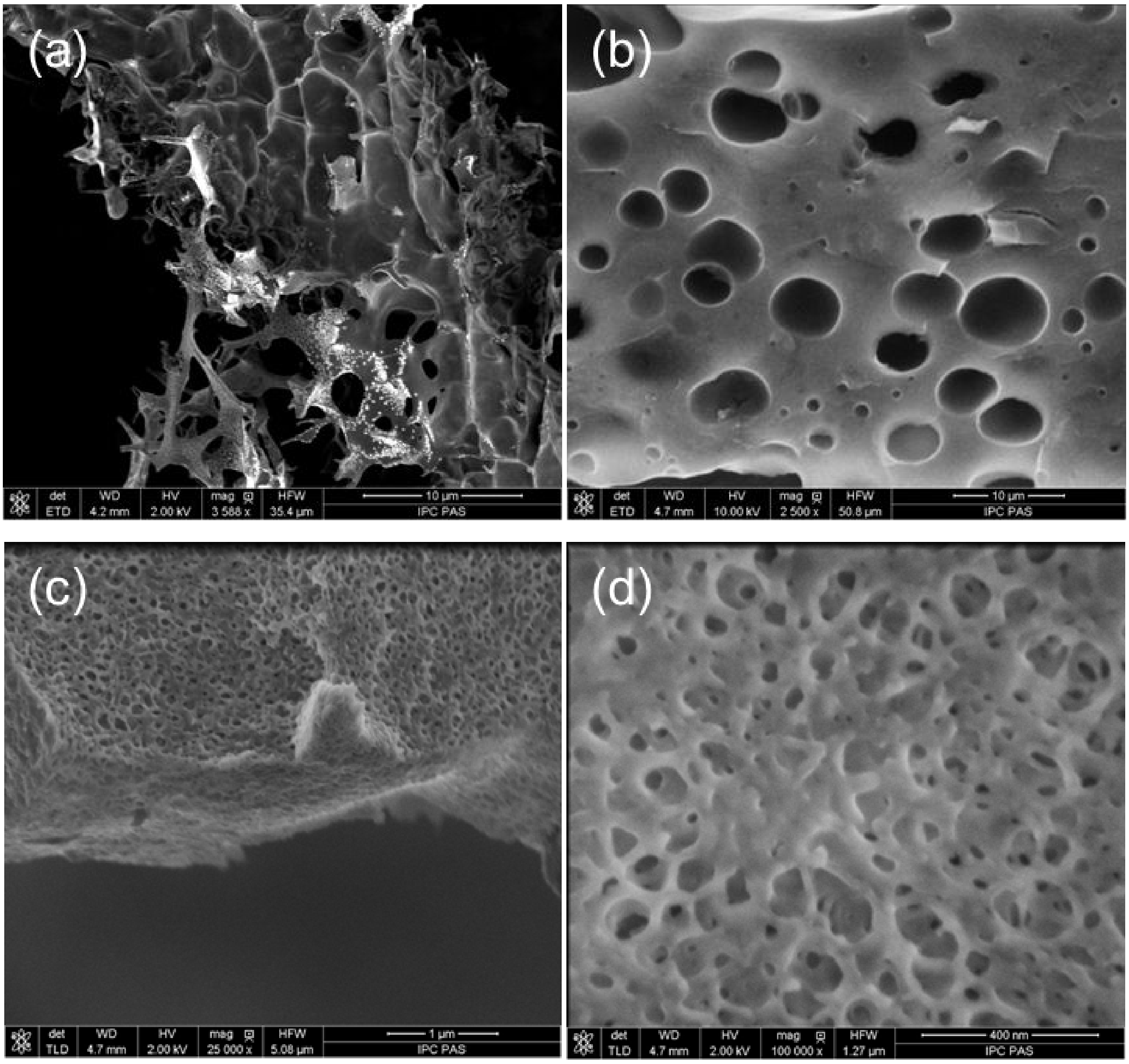

Figure S6. *Scanning electron microscopy (SEM) images of lyophilized hydrogels. (a) Laterally sectioned of GelMA/PEDOT:PSS (G/P) hydrogel showing heterogeneous distribution of PEDOT:PSS aggregates (bright regions). (b) GelMA/PEDOT:PSS/PEDOT-TMA:TOS (G/P/P-TMA) imaged without metal sputtering at 10 kV, confirming intrinsic conductivity. (c) Bottom surface of G/P/P-TMA hydrogel, demonstrating uniform microstructure and absence of conductive biofilm formation, confirming bulk-mediated conductivity. (d) High-magnification view of G/P/P-TMA revealing bimodal porosity with macropores (5–10 µm, panel b) and micropores (10–200 nm).*

6. Supplementary Figure S7. C2C12 Live/Dead assay

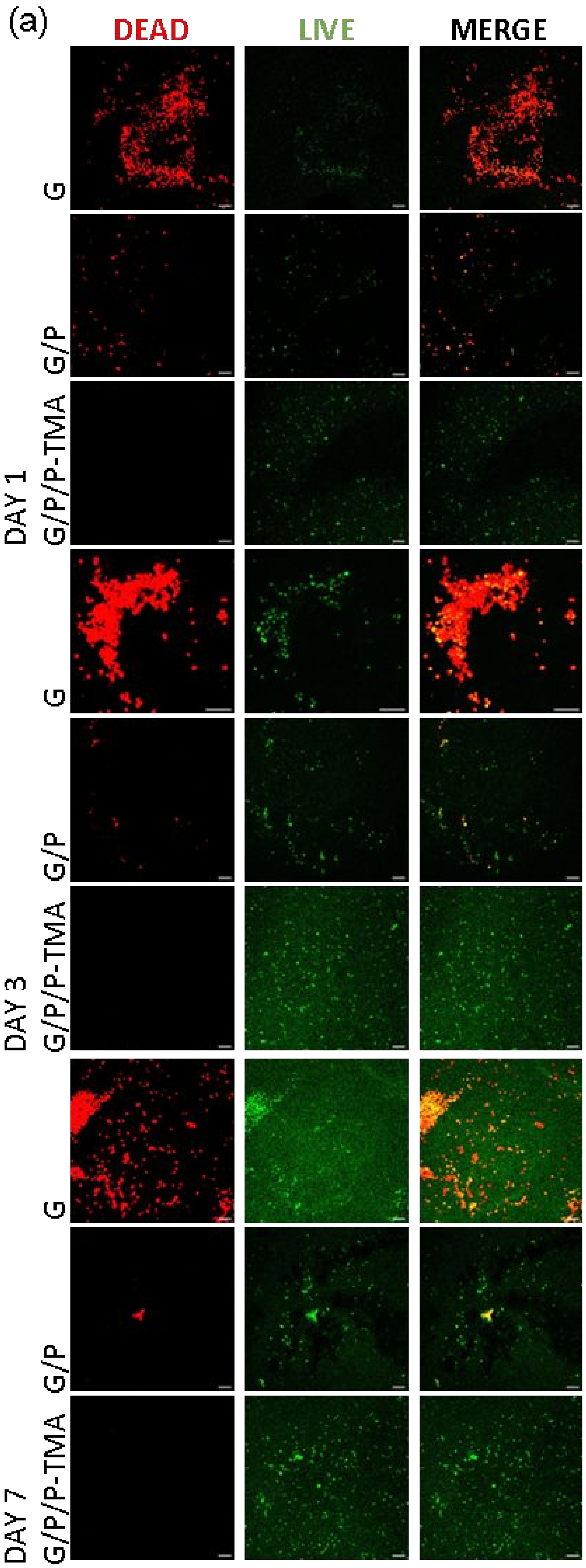

(b)

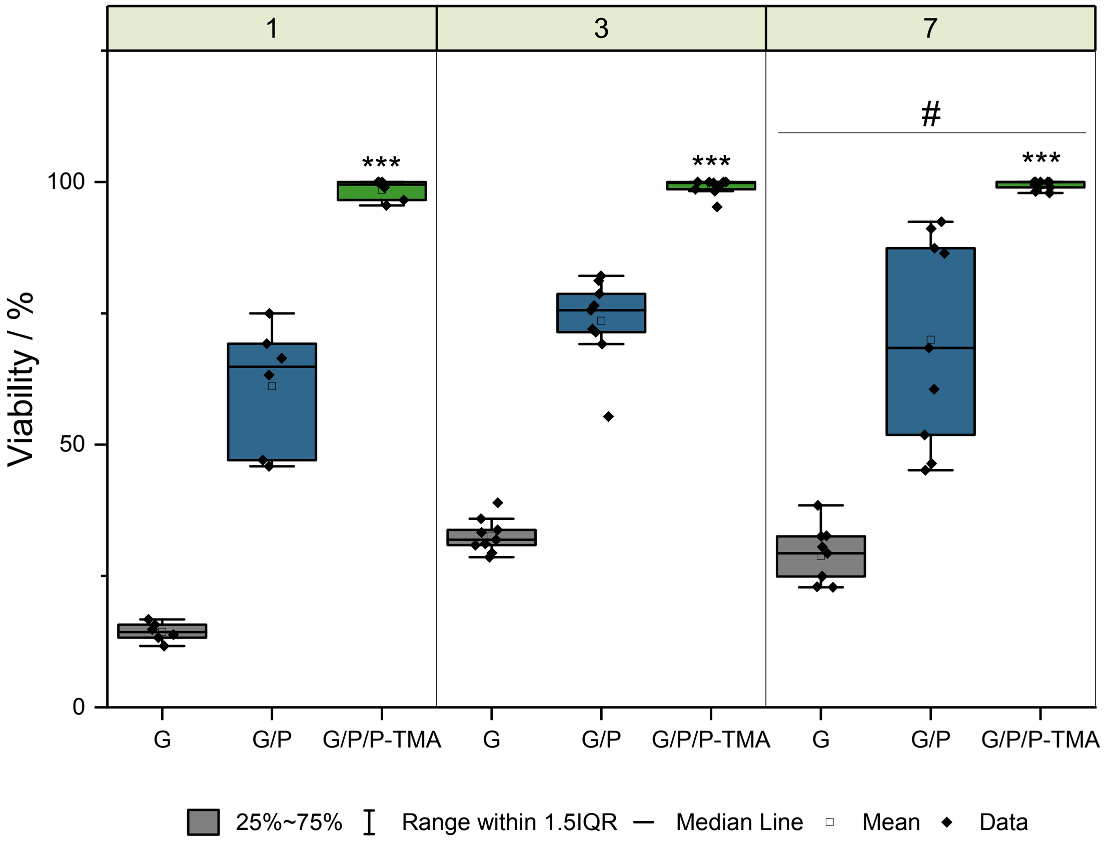

Figure S7. *Cytocompatibility assessment of C2C12 cells laden in hydrogels. (a) Live/dead confocal images (green: live cells; red: dead cells; scale: 100 μm). (b) Viability quantification (mean ± SD, n=3). ***p<0.001 vs. all controls; #p<0.01 vs. Day 1 within same group.*

7. Supplementary Table S2. Different approaches to GelMA + CP composition and resulting properties

Table S2. *Summary of the literature on the electrochemical performance of conducting hydrogel in comparison to this work. (Gelatin methacryloyl (GelMA), poly(3,4-ethylenedioxytiophene) (PEDOT), polystyrene sulfonate (PSS), poly(3,4-ethylenedioxytiophene)-tetramethacrylate (PEDOT-TMA), p-toluenesulfonate (TOS), poly(ethylene glycol) diacrylate (PEGDA), organic electrochemical transistor (OECT)).*

**Study**	**System/composition**	**Key electro-physical metrics**	**Biological focus**
A. R. Spencer et al.[3]	Cold-fish skin GelMA (7%) + PEDOT:PSS (0.1–0.3%)	Conductivity: 5.0×10^{-5} S/m	C2C12 myoblast viability (high viability at 0.1%; reduced at 0.3%)
F. Tan et al.[4]	Cold-fish skin GelMA (5%) + PEDOT:PSS (0.15%)	Conductivity: 0.30–0.43 S/m	Cell compatibility (unspecified cell type)
Y. Zhang et al.[5]	Mammalian GelMA (Type B, 6%) + PEDOT:PSS (0.005–0.015%)	Conductivity: 0.065–0.134 S/m	Neural stem cells (*in vitro* / *in vivo* ischemic model)
D. Testore et al.[6]	Cold-fish skin gelatine + PEGDA (photo-curable hydrogel) + PEDOT:PSS (0.1–0.5%)	Conductivity: 1.2–1.3 × 10^{-5} S/m (dry); Impedance (1 Hz): 1.8 MΩ (with 0.5% PEDOT)	Human cardiac fibroblasts viability and adhesion
**This Work**	Cold-fish skin GelMA (4%) + PEDOT:PSS (0.23%) + PEDOT-TMA:TOS (0.23%)	Conductivity: 2.9 S/m; OECT performance: $g_m = 2.13 \pm 0.86$ mS, $V_{th} = 0.216 \pm 0.031$ V, $I_{ON}/I_{OFF} \approx 157$; impedance reduced by five orders of magnitude *vs.* GelMA only	High C2C12 cell viability (> 98%) and homogeneous dispersion verified via SEM

References

(1) Fairbanks, B. D.; Schwartz, M. P.; Bowman, C. N.; Anseth, K. S. Photoinitiated Polymerization of PEG-Diacrylate with Lithium Phenyl-2,4,6-Trimethylbenzoylphosphinate: Polymerization Rate and Cytocompatibility. *Biomaterials* **2009**, *30* (35), 6702–6707.

(2) Filipiak, M. S.; Wróblewska, M.; Suranglikar, M.; Stratmann, L.; Heery, B. Facilitating Field-Effect Transistor Based (Bio)Sensor Research with a Miniaturized Bipotentiostat. Chemistry May 18, **2022**.

(3) Spencer, A. R.; Primbetova, A.; Koppes, A. N.; Koppes, R. A.; Fenniri, H.; Annabi, N. Electroconductive Gelatin Methacryloyl-PEDOT:PSS Composite Hydrogels: Design, Synthesis, and Properties. *ACS Biomater. Sci. Eng.* **2018**, acsbiomaterials.8b00135.

(4) Tan, F.; Li, X.; Li, X.; Xu, M.; Shahzad, K. A.; Hou, L. GelMA/PEDOT:PSS Composite Conductive Hydrogel-Based Generation and Protection of Cochlear Hair Cells through Multiple Signaling Pathways. *Biomolecules* **2024**, *14* (1), 95.

(5) Zhang, Y.; Zhang, M.; Zhang, R.; Liu, H.; Chen, H.; Zhang, X.; Li, C.; Zeng, Q.; Chen, Y.; Huang, G. Conductive GelMA/PEDOT: PSS Hybrid Hydrogel as a Neural Stem Cell Niche for Treating Cerebral Ischemia-Reperfusion Injury. *Front. Mater.* **2022**, *9*, 914994.

(6) Testore, D.; Zoso, A.; Kortaberria, G.; Sangermano, M.; Chiono, V. Electroconductive Photo-Curable PEGDA-Gelatin/PEDOT:PSS Hydrogels for Prospective Cardiac Tissue Engineering Application. *Front. Bioeng. Biotechnol.* **2022**, *10*, 897575.
